# Unusual presentation of fatal disseminated varicella zoster virus infection in a patient with lupus nephritis: a case report

**DOI:** 10.1186/s12879-020-05254-6

**Published:** 2020-07-23

**Authors:** Veronica Vassia, Alessandro Croce, Paolo Ravanini, Monica Leutner, Chiara Saglietti, Stefano Fangazio, Marco Quaglia, Carlo Smirne

**Affiliations:** 1 Department of Translational Medicine, DiMet, Università del Piemonte Orientale, via Solaroli 17, 28100 Novara, Italy; 2 Laboratory of Molecular Virology, AOU Maggiore della Carità, Novara, Italy; 3 Histopathology Unit, AOU Maggiore della Carità, Novara, Italy

**Keywords:** Varicella zoster virus, Herpesviruses, Systemic lupus erythematosus, Immunosuppression, Multiple virus reactivations, Latent virus infections, Mycophenolate mofetil, Steroid therapy

## Abstract

**Background:**

The risk of life-threatening complications, such as visceral disseminated varicella zoster virus (VZV) infection, is greater in immunosuppressed individuals, such as systemic lupus erythematosus (SLE) patients.

**Case presentation:**

Here, a case is reported of a Caucasian woman diagnosed with lupus nephritis and anti-phospholipid syndrome, who was subjected to mycophenolate mofetil and high-dose steroid remission-induction therapy. Two months later she developed abdominal pain followed by a fatal rapid multi-organ failure. As no typical skin rashes were evident, death was initially attributed to catastrophic anti-phospholipid syndrome. However, autopsy and virological examinations on archival material revealed a disseminated VZV infection.

**Conclusions:**

Overall, this case highlights the importance of having a high clinical suspicion of fatal VZV infections in heavily immunosuppressed SLE patients even when typical signs and symptoms are lacking.

## Background

Varicella-zoster virus (VZV), a member of the family of herpesviridae, remains latent in dorsal roots and autonomic ganglia after a primary infection, but it can always reactivate leading to a secondary infection, which is usually characterized by skin rash and acute neuritis. In some cases, more severe complications may occur especially in immunocompromised patients, where multi-organ involvement can develop with manifestations such as encephalitis, aseptic meningitis, pneumonia and hepatitis [1].

Here, a case is reported of fatal visceral disseminated VZV in a patient affected by systemic lupus erythematosus (SLE) and anti-phospholipid syndrome (APS) treated with mycophenolate mofetil (MMF) and high-dose glucocorticoids.

## Case presentation

A 49-year-old Caucasian woman affected by SLE and APS presented to the Emergency Department complaining of acute onset of abdominal pain. She denied fever, nausea or vomit. Two months earlier, the patient, with no previous medical history, had been hospitalized for deep vein thrombosis. Her vaccination history was as follows: diphtheria, polio, smallpox and tetanus; she had never been vaccinated for chickenpox, measles, mumps or rubella. During that period, she had been diagnosed with both SLE —positive homogeneous antinuclear antibody titer of 1:320 with a homogeneous pattern— and APS —positive lupus anticoagulant—, with multi-organ dysfunction consisting of lupus nephritis with nephrotic syndrome, lung serositis, hemolytic anemia and arthritis. Consequently, the patient had been treated with MMF (1.5 g qd) and prednisone (50 mg qd) by nephrologists. On hospital admission, vitals were normal except for heart rate at 120 bpm. Physical examination revealed petechiae at thorax and limbs. Complete blood count showed low lymphocyte [0.50 × 10^9^/L] and platelet (58 × 10^9^/L) counts. Other serum abnormal laboratory data included decreased levels of immunoglobulin (Ig) G [118 mg/dL (NR:700–1600)] and increased values of aspartate aminotransferase (AST), alanine aminotransferase (ALT) [22x and 13x upper limit of normal (ULN), respectively] and lactate dehydrogenase (LDH) [13x ULN]. Activated partial thromboplastin time [1.5x ULN] and international normalized ratio (INR) [5.9] were prolonged, whereas renal function was normal. At peripheral blood smear, a number of echinocytes and 2–3 schistocytes/high-power field were detected; platelets were normal-sized but decreased (5/high-power field).

All these findings led to the suspicion of catastrophic APS (CAPS). Therefore, methylprednisolone (1 g qd) was started, but the patient’s conditions worsened dramatically as blood tests revealed persistent decrease in platelet count [23 × 10^9^/L] and substantial increase in INR [7.75], LDH [25x ULN] and D-dimer [71x ULN], consistent with a possible disseminated intravascular coagulation (DIC). Therefore, platelet transfusions and fresh frozen plasma were administered. Later on, plasmapheresis and immunoglobulin infusion became necessary. The patient began to show signs of multiple organ failure (MOF) such as acute kidney injury, increasing elevation of liver function tests, glycemia and troponin I, appearance of confusion and, finally, respiratory distress that required intubation. During the procedure, pseudo-membranes, white exudates and diffuse petechiae were detected in the pharynx (consistent with a possible infective exudative pharyngitis). Later on, hypotension and acute onset anemia appeared, and the patient eventually died a few hours later.

**Fig. 1:**
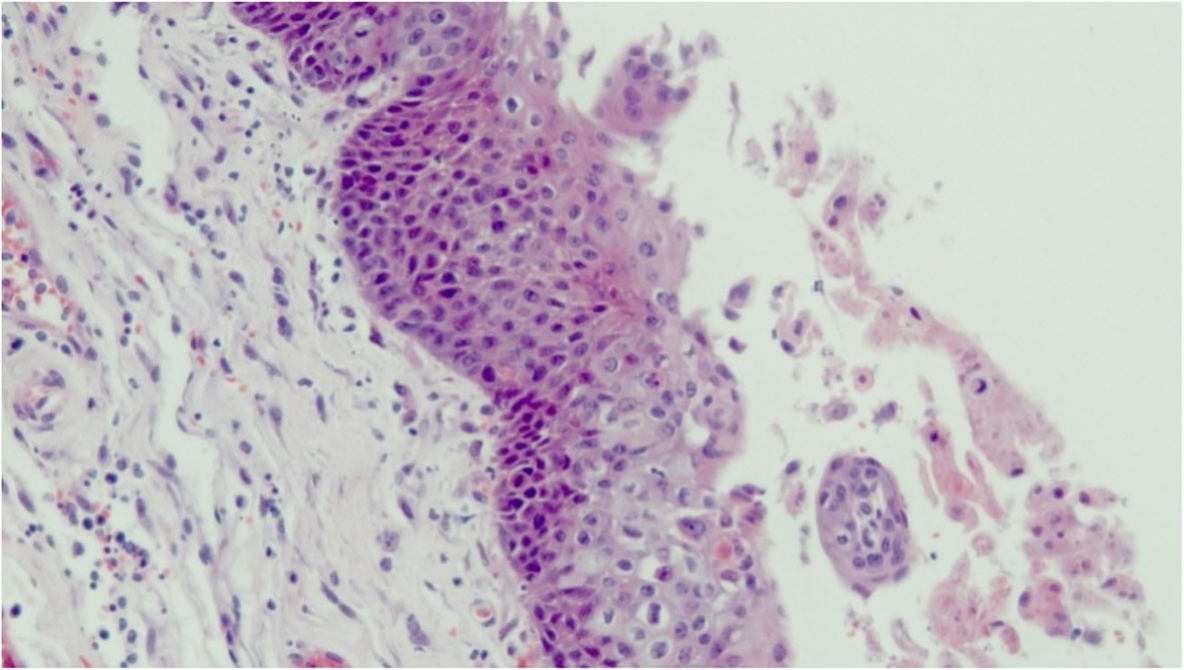
Histological findings at autopsy. Pharyngeal mucosa showing acantholytic keratinocytes with intranuclear inclusions (hematoxylin-eosin staining, magnification × 200)

**Fig. 2:**
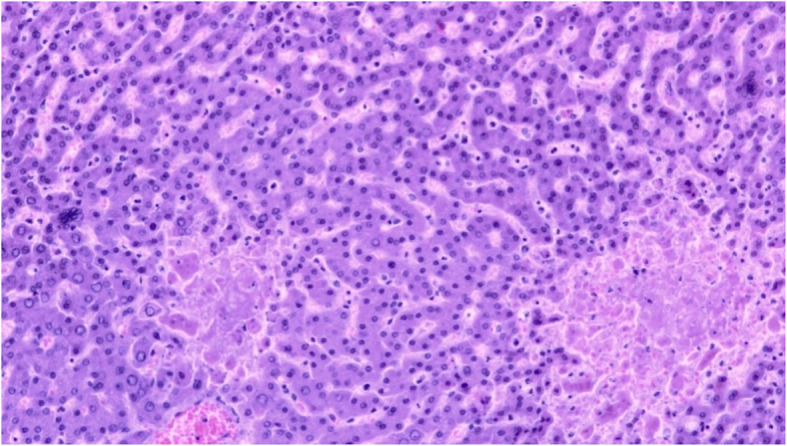
Histological findings at autopsy. Hepatic parenchyma with foci of coagulative necrosis, multinucleated hepatocytes (center left and top right) and ground-glass nuclei (hematoxylin-eosin staining, magnification × 200)

At autopsy, diffuse skin petechiae were present. No significant small vessel occlusions could be observed. Pharyngeal mucosa showed ulcerative lesions associated with cytopathic effects of the squamous epithelium (including acantholysis, intranuclear inclusions and cytoplasmic vacuolization), which were suggestive of viral infection (Fig. 1). The liver parenchyma contained areas of coagulative and hemorrhagic necrosis, and hepatocyte nuclei had a diffuse ground-glass appearance, suspicious for viral inclusions. Moreover, occasional multinucleated hepatocytes were observed (Fig. 2). No other significant alterations were present. Being the morphological picture at pharyngeal and liver level consistent with herpesvirus (HV) infection [2], immunohistochemistry was performed with locally available antibodies [i.e. herpes simplex virus 1 (HSV-1) and 2 (HSV-2) and cytomegalovirus (CMV)], but it resulted negative both in liver and in pharynx.

**Fig. 3:**
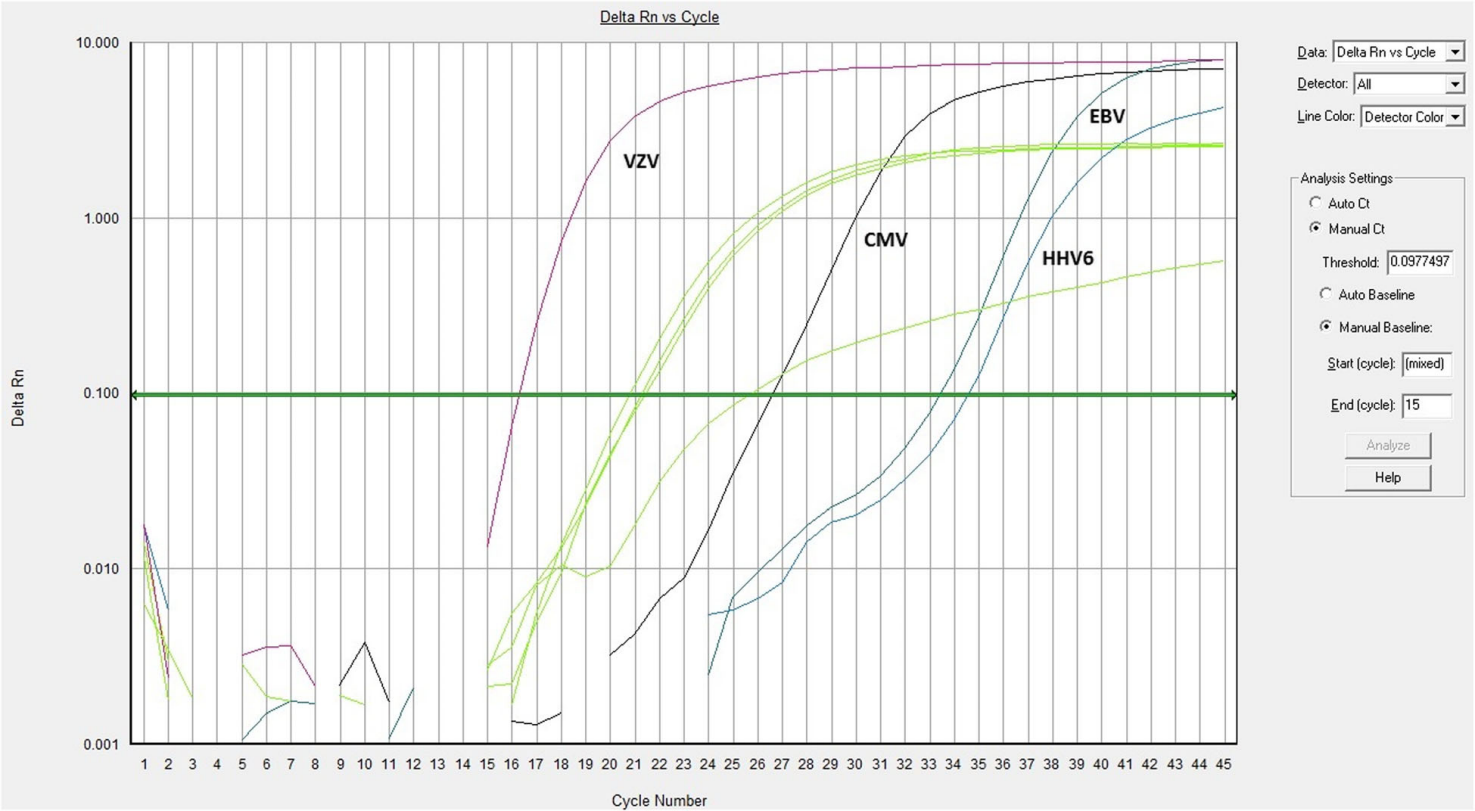
Amplification curves of herpesviruses quantitative real-time polymerase-chain reaction assays. On the x-axis the cycle numbers are represented. On the y-axis the fluorescence signals are reported on a logarithmic scale; Rn is the fluorescence of the reporter dye divided by the fluorescence of the passive reference dye. Green lines represent the four internal controls. The other four curves represent the viral targets as indicated in the Figure (i.e. VZV, CMV, EBV, and HHV-6). The horizontal central line is the threshold line

In light of these findings and after a multidisciplinary discussion of the case, it was opted to perform virological tests taking in due account the interpretative limitations related to hypogammaglobulinemia and quantitative real-time polymerase-chain reaction assays (PCR; ELITe MGB Kits, ELITech, France) for HV on the plasma samples that had been harvested on the day of death. The most relevant data was a strong VZV positivity (19,600,000 copies/mL) in the absence of concomitant immune activation [IgM index = 0.3 (cut-off > 1); IgG < 10 mIU/mL (cut-off > 150)]. Furthermore, it was found positivity for CMV [serological pattern of reactivation with DNA at 3240 IU/ml], Epstein–Barr virus (EBV) [serological pattern of reactivation with detectable DNA below the lower limit of quantitation (LLQ) of 256 IU/mL] and human herpesvirus 6 (HHV-6) [DNA below the LLQ of 350 IU/mL], whereas samples were negative for HSV-1 and HSV-2 DNA (Fig. 3).

## Discussion and conclusions

VZV infection may develop as a primary infection or reactivation, the latter one being usually characterized by skin rash and acute neuritis. Nevertheless, immunocompromised patients may display more severe and atypical manifestations, such as encephalitis, aseptic meningitis, pneumonia and hepatitis, along with disseminated visceral and cutaneous involvement [1]. These manifestations frequently represent a diagnostic challenge, as the clinical picture might be insidious, leading to a difficulty in the administration of a prompt and appropriate treatment [3]. Therefore, it appears mandatory to take into account unusual signs and symptoms, especially since these subjects are at higher risk for the rare, but severe, disseminated VZV infection. Amongst immunosuppressed subjects, SLE patients have been demonstrated to have a particularly high incidence of VZV infection [4, 5]. However, the determinants of this positive association have so far remained unclear, even though a lack of cellular and/or humoral immunity, such as lymphopenia, may be playing a major role [6–8]. Another explanation may be that the use of immunosuppressant drugs is itself a risk factor for VZV. Indeed, the use of combination therapies consisting of MMF and high-dose glucocorticoids has been shown to increase VZV susceptibility [9]. This aspect is particularly relevant to our case. Nevertheless, it is noteworthy that, to be the best of our knowledge, large studies examining the incidence of infections in patients with rheumatic illness treated with MMF are still lacking, so that generalizations can not yet be made. Taking into account these limitations, only a few studies suggest an increased incidence of infectious diseases in such patients [10, 11], as MMF is generally considered a safe drug compared to other immunosuppressive treatments, such as cyclophosphamide [12]. So, even if a MMF role in facilitating viral infections is strongly suspected, a regular monitoring of the presence of VZV in rheumatic patients treated with MMF is not yet recommended.

One of the most recent contributions from the Literature on this topic comes from Habuka et al. who recently described a dramatic case of disseminated VZV infection in a patient with lupus nephritis treated with the aforementioned combination regimen, not only highlighting again the possible harmful effect of immunosuppressive drug therapy, but also suggesting a possible connection between being Asian and experiencing worse outcomes [13]. Although these elements make that case similar to ours, here it is shown an unprecedented case in a Caucasian subject. Moreover, our patient presented with a rarely found combination of disseminated VZV infection and MOF. Another peculiarity of our case is that the patient did not show any signs or symptoms suggestive of VZV infection, when this latter is usually accompanied by cutaneous manifestations, which help make a correct diagnosis. The only complaint reported by the patient was in fact abdominal pain. This symptom is generally considered uncommon and it has always been reported as being concomitant with or, at least, preceding skin manifestations [14]; it is generally considered to occur due to direct viral infection of the enteric nervous system, from at least two different pathways: viremia, in which circulating T cells carry VZV, and axonal transport from dorsal root ganglia [14, 15]. Coming back to our patient, it is possible that she did not have the time to manifest typical skin signs. Not surprisingly, the initial diagnosis failed to recognize viral infection. The differential diagnosis was in fact between acute DIC and CAPS. However, since a clear confirmation of small vessel occlusion was lacking at autopsy, the final cause of death was attributed to MOF secondary to severe sepsis, the latter one being likely also the cause of the severe progressive thrombocytopenia.

Another distinctive feature of this case is that, while autopsy revealed signs of viral infection almost exclusively in the pharynx and liver, blood PCR assays on archival material showed an extremely high viral VZV load. Unfortunately, it is not known how much time went by from the first infection/reactivation to sample collection. In any case, considering that the virological tests were performed 3 weeks later, it is tempting to speculate that there should have been an even higher viral load at the collection time. Thus, VZV serology seems to indicate a recent first infection to which the patient was not able to react, probably due to a severe immunosuppression state, as suggested by hypogammaglobulinemia and lymphopenia (although, at least for the latter one, viral infection cannot be excluded as a contributory cause).

In this patient, the microbiological pattern was further complicated by the occurrence of multiple HV infections concomitant with VZV. These coreactivations are usually associated with poorer outcomes [16, 17]. Among the most commonly reactivated HV, CMV and EBV and, to a lesser extent, also HSV-1, HHV-6 and VZV have been reported [18, 19]. Alternatively, reactivated HV can directly transactivate other latent viruses. As an example, it is known that HHV-6A can reactivate EBV through a BamHI Z fragment leftward open reading frame (BZFL)-1-dependent mechanism, or through the activation of the Zebra promoter by a cyclic AMP-responsive element [20, 21]. Thus, it is apparent that being able to differentiate between clinically significant and latent viral burden is of the utmost importance when dealing with immunosuppressed patients. In this regard, quantitative PCR assays can provide valuable help. In our patient, although the CMV viral load was not as high as that of VZV, it reached threshold values usually recommended for the initiation of preemptive therapy. In contrast, EBV and HHV-6 viremias were below the LLQ, so it was not possible to determine whether they corresponded to true latent virus reactivations, as it was suggested by the concomitant serological responses.

In conclusion, our investigation calls for increased awareness about the unpredictable complications caused by reactivations of specific latent infections under strong immunosuppression: prompt clinical diagnosis might be challenging, so early viral detection is highly recommended [3]. This is because a high index of suspicion on the part of the clinician to monitor for the presence of VZV in MMF-treated patients could result in appropriate timely treatment with acyclovir and better patient outcomes.

## Data Availability

All data generated during the survey of this case are included in this published article. The medical file and the histological archive of the patient are available from the corresponding author on reasonable request.

## Abbreviations

VZV: Varicella-zoster virus
HV: Herpesvirus
SLE: Systemic lupus erythematosus
APS: Anti-phospholipid syndrome
MMF: Mycophenolate mofetil
Ig: Immunoglobulin
AST: Aspartate aminotransferase;
ALT: Alanine aminotransferase
ULN: Upper limit of normal
LDH: Lactate dehydrogenase
INR: International normalized ratio
CAPS: Catastrophic anti-phospholipid syndrome
DIC: Disseminated intravascular coagulation
MOF: Multiple organ failure
HSV-1: Herpes simplex virus 1
HSV-2: Herpes simplex virus 2
CMV: Cytomegalovirus
PCR: Polymerase-chain reaction
EBV: Epstein–Barr virus
LLQ: Lower limit of quantitation (LLQ)
HHV-6: Human herpesvirus 6
BZFL: BamHI Z fragment leftward open reading frame

## References

[1] Gnann JW (2002). Varicella-zoster virus: atypical presentations and unusual complications. J Infect Dis.

[2] Nikkels AF, Delvenne P, Sadzot-Delvaux C, Debrus S, Piette J, Rentier B (1996). Distribution of varicella zoster virus and herpes simplex virus in disseminated fatal infections. J Clin Pathol.

[3] Lewis D, Schlichte M, Dao H (2017). Atypical disseminated herpes zoster: management guidelines in Immunocompromised patients. Cutis.

[4] Borba EF, Ribeiro AC, Martin P, Costa LP, Guedes LK, Bonfá E (2010). Incidence, risk factors, and outcome of herpes zoster in systemic lupus erythematosus. J Clin Rheumatol.

[5] Sayeeda A, Al Arfaj H, Khalil N, Al Arfaj AS. Herpes zoster infections in SLE in a University Hospital in Saudi Arabia: risk factors and outcomes. Autoimmune Dis. 2010. 10.4061/2010/174891. 10.4061/2010/174891 PMC2989732 21152215

[6] Rondaan C, de Haan A, Horst G, Hempel JC, van Leer C, Bos NA (2014). Altered cellular and humoral immunity to varicella-zoster virus in patients with autoimmune diseases. Arthritis Rheumatol.

[7] Park HB, Kim KC, Park JH, Kang TY, Lee HS, Kim TH (2004). Association of reduced CD4 T cell responses specific to varicella zoster virus with high incidence of herpes zoster in patients with systemic lupus erythematosus. J Rheumatol.

[8] Chen D, Li H, Xie J, Zhan Z, Liang L, Yang X (2017). Herpes zoster in patients with systemic lupus erythematosus: clinical features, complications and risk factors. Exp Ther Med.

[9] Chakravarty EF, Michaud K, Katz R, Wolfe F (2013). Incidence of herpes zoster among patients with systemic lupus Erythematosus. Lupus.

[10] Meirinhos T, Mariz E, Castro Ferreira I, Neto R, Pereira E, Costa L. AB0424 Retrospective Evaluation of Mycophenolate Mofetil Infectious Side Effects on Lupus Nephritis Patients. Ann Rheum Dis. 2016. 10.1136/annrheumdis-2016-eular.5961.

[11] Kingdon EJ, McLean AG, Psimenou E, Davenport A, Powis SH, Sweny P (2001). The safety and efficacy of MMF in lupus nephritis: a pilot study. Lupus.

[12] Henderson L, Masson P, Craig JC, Flanc RS, Roberts MA, Strippoli GF, et al. Treatment for lupus nephritis. Cochrane Database Syst Rev. 2012. 10.1002/14651858.CD002922.pub3. 10.1002/14651858.CD002922.pub3 23235592

[13] Habuka M, Wada Y, Kurosawa Y, Yamamoto S, Tani Y, Ohashi R (2018). Fatal visceral disseminated varicella zoster infection during initial remission induction therapy in a patient with lupus nephritis and rheumatoid arthritis-possible association with mycophenolate mofetil and high-dose glucocorticoid therapy: a case report. BMC Res Notes.

[14] Okuma HS, Kobayashi Y, Makita S, Kitahara H, Fukuhara S, Munakata W (2016). Disseminated herpes zoster infection initially presenting with abdominal pain in patients with lymphoma undergoing conventional chemotherapy: a report of three cases. Oncol Lett.

[15] Balkis MM, Ghosn S, Sharara AI, Atweh SF, Kanj SS (2009). Disseminated varicella presenting as acute abdominal pain nine days before the appearance of the rash. Int J Infect Dis.

[16] Curley MJ, Hussein SA, Hassoun PM (2002). Disseminated herpes simplex virus and varicella zoster virus coinfection in a patient taking thalidomide for relapsed multiple myeloma. J Clin Microbiol.

[17] Lopez Roa P, Hill JA, Kirby KA, Leisenring WM, Huang ML, Santo TK (2015). Coreactivation of human Herpesvirus 6 and Cytomegalovirus is associated with worse clinical outcome in critically ill adults. Crit Care Med.

[18] Grinde B. Herpesviruses: latency and reactivation - viral strategies and host response. J Oral Microbiol. 2013. 10.3402/jom.v5i0.22766. 10.3402/jom.v5i0.22766 PMC3809354 24167660

[19] Weinberg A, Bloch KC, Li S, Tang YW, Palmer M, Tyler KL (2005). Dual infections of the central nervous system with Epstein-Barr virus. J Infect Dis.

[20] Cuomo L, Angeloni A, Zompetta C, Cirone M, Calogero A, Frati L (1995). Human herpesvirus 6 variant a, but not variant B, infects EBV-positive B lymphoid cells, activating the latent EBV genome through a BZLF-1-dependent mechanism. AIDS Res Hum Retrovir.

[21] Flamand L, Menezes J (1996). Cyclic AMP-responsive element-dependent activation of Epstein-Barr virus zebra promoter by human herpesvirus 6. J Virol.

